# Efficient Terahertz Wide-Angle NUFFT-Based Inverse Synthetic Aperture Imaging Considering Spherical Wavefront

**DOI:** 10.3390/s16122120

**Published:** 2016-12-14

**Authors:** Jingkun Gao, Bin Deng, Yuliang Qin, Hongqiang Wang, Xiang Li

**Affiliations:** School of Electronic Science and Engineering, National University of Defense Technology, Changsha 410073, China; oscar92923@163.com (J.G.); qinyuliang@nudt.edu.cn (Y.Q.); oliverwhq@tom.com (H.W.); lixiang01@vip.sina.com (X.L.)

**Keywords:** spherical wavefront, wide-angle, terahertz imaging, synthetic aperture imaging

## Abstract

An efficient wide-angle inverse synthetic aperture imaging method considering the spherical wavefront effects and suitable for the terahertz band is presented. Firstly, the echo signal model under spherical wave assumption is established, and the detailed wavefront curvature compensation method accelerated by 1D fast Fourier transform (FFT) is discussed. Then, to speed up the reconstruction procedure, the fast Gaussian gridding (FGG)-based nonuniform FFT (NUFFT) is employed to focus the image. Finally, proof-of-principle experiments are carried out and the results are compared with the ones obtained by the convolution back-projection (CBP) algorithm. The results demonstrate the effectiveness and the efficiency of the presented method. This imaging method can be directly used in the field of nondestructive detection and can also be used to provide a solution for the calculation of the far-field RCSs (Radar Cross Section) of targets in the terahertz regime.

## 1. Introduction

Terahertz waves are of short wavelength, moderate penetrability and low electron energy [1]. Due to these special characteristics, terahertz waves are thought to do no damage to biological tissues and terahertz radar can achieve a larger signal bandwidth, higher range resolution and higher Doppler resolution more easily. On the contrary, other than these advantages, there is a relatively stronger atmosphere attenuation to terahertz waves. Thus, its propagation distance is not as long as that of lower bands and the operating range of the terahertz radar is confined [2].

With the development of the terahertz radar system, the corresponding application technologies including inverse synthetic aperture radar (ISAR) imaging also thrive [3,4,5,6,7,8]. The expected detection range of the terahertz radar can reach several kilometers within the atmospheric layer. However, confined by the recent level of transmission power, the typical detection range of the terahertz radar is about several to tens of meters. From the current terahertz ISAR imaging results, we can see that the shorter wavelength and the larger bandwidth non-accidentally lead to higher resolution compared to the traditional microwave band’s radar images [9,10]. 

At terahertz frequencies, due to the short wavelength, high spatial resolution and limited operating range, the influence of the spherical wavefront on the ISAR images can become more obvious. In the current reports on the terahertz ISAR imaging results, researchers usually employ the plane wave approximation whose validity is determined by the size of the target, the distance between the radar and the target, the range of the rotation angle and so on. In most of the recent works, the spherical wavefront effects are neglected or the experimental configurations are designed to alleviate this effect. 

In lower bands, such as microwaves and millimeter waves, there have been several studies of SAR/ISAR on wavefront curvature compensation and wide-angle imaging (e.g., circular SAR) problems. One of the most powerful methods is the back-projection (BP) algorithm [11,12], though it suffers from a heavy computational burden. In SAR, several methods have been proposed to solve the wavefront curvature problem, such as the tiered sub-aperture algorithm (TSA) [13], space-variant postfiltering (SVPF) [14,15] and widefield polar format algorithm (WPFA) [16]. However, these methods are usually under the assumption that the radar platform is following an expected linear trajectory and becomes invalid under a circular trajectory. Also, these methods usually use sub-apertures or sub-images to make approximations, which impacts the accuracy of the compensation. In circular synthetic aperture radar (CSAR), there are also several reports on the wavefront curvature problem [17], and piecewise planar approximation [18] and spherical wave decomposition [19,20] techniques are utilized. However, the accuracy and computational efficiency of these methods are still not satisfactory. In wide-angle ISAR, the spherical compensation method based on the circular convolution technique is proposed [21,22,23]. However, at terahertz frequencies, no conclusions on whether it is valid have been made, and the efficiency of their image reconstruction method can still be promoted. 

In this paper, we focus on terahertz ISAR imaging under the spherical wave (or near-field) condition and demonstrate a fast and accurate wide-angle ISAR imaging method which combines the circular convolution and nonuniform fast Fourier transform (NUFFT) techniques. The advantages of this method include: (1) Accuracy. Different from some typical –aperture- or sub-image-based wavefront curvature compensation methods in SAR/ISAR, no sub-aperture or sub-image approximations and no Taylor approximation are introduced in this method; (2) Efficiency. The spherical wave effects are calibrated in the spatial wavenumber domain, and the image is focused all at once without post-processing. As the computation for wavefront compensation and image reconstruction mainly comes from fast Fourier transform (FFT) and NUFFT manipulations, this method is far less time-consuming than the convolution back-projection (CBP) algorithm; (3) No rotation angle restrictions. Unlike the rotation-angle–confined near-field imaging method [24], this method is not limited by the scope of the rotation angle. The wavefront curvature can be calibrated for arbitrary rotation angles within (0,2π). 

Based on the near-field imaging method in this paper, terahertz ISAR imaging can be directly applied to industrial nondestructive testing, security checks and so on. It also provides a feasible solution for the calculation of the far-field RCSs (Radar Cross Section) of targets in the terahertz regime. To the best of our knowledge, this is the first time that a fast and accurate wide-angle imaging method considering wavefront curvature has been demonstrated at terahertz frequencies. 

## 2. Model and Methods

**Figure 1:**
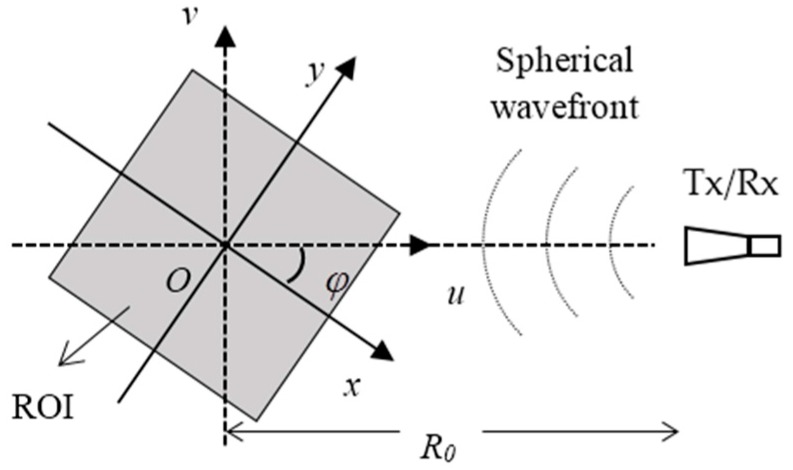
Geometric model and basic parameters.

Figure 1 illustrates the basic geometric model which the following studies build upon. xOy is the target coordinate, uOv is the radar coordinate, and they share the same origin. $R_{0}$ is a constant that represents the distance between the origin O and the radar antenna; φ represents the angle from axis x to axis u. The radar transmits an linear frequency modulation (LFM) signal and receives the echo waves in a dechirping manner, and the reference distance selected by the radar system is zero. Suppose the target is rotating at a uniform angular speed, and the echo signal received by the radar can be written as:

(1) $$\begin{array}{l} S\left(k,\varphi \right)=\iint_{x,y}f\left(x,y\right)\\ \;\cdot \exp\left(2jk\sqrt{{\left(R_{0}\cos\varphi -x\right)}^{2}+{\left(R_{0}\sin\varphi -y\right)}^{2}}\right)dxdy \end{array}$$

where f(x,y) represents the target function, S(k,φ) is the echo signal in the spectral domain and we neglect the amplitude attenuation term that has less impact on the algorithm derivations. Further, $k=\frac{2\pi f}{c}=\frac{2\pi }{\lambda }$ stands for the spatial wavenumber of the electromagnetic wave. The integral kernel of Equation (1) is the spherical wave term. By using the plane wave expansion technology, it can be expressed by the following integration [25]:

(2) $$\begin{array}{l} \exp\left(2jk\sqrt{{\left(R_{0}\cos\varphi -x\right)}^{2}+{\left(R_{0}\sin\varphi -y\right)}^{2}}\right)\\ \approx \int_{-\pi }^{\pi }\exp\left(2jk\left(\cos\alpha \left(R_{0}\cos\varphi -x\right)+\sin\alpha \left(R_{0}\sin\varphi -y\right)\right)\right)d\alpha \end{array}$$

Substitute Equation (2) into Equation (1) and we can obtain:

(3) $$\begin{array}{l} S\left(k,\varphi \right)=\iint_{x,y}f\left(x,y\right)\\ \cdot \int_{-\pi }^{\pi }\exp\left(2jk\left(\cos\alpha \left(R_{0}\cos\varphi -x\right)+\sin\alpha \left(R_{0}\sin\varphi -y\right)\right)\right)d\alpha dxdy\\ =\int_{-\pi }^{\pi }\iint_{x,y}f\left(x,y\right)\exp\left(-2jkx\cos\alpha -2jky\sin\alpha \right)dxdy\\ \;\;\cdot \exp\left(2jkR_{0}\cos\left(\varphi -\alpha \right)\right)d\alpha \end{array}$$

For convenience, we define

(4) $$F\left(k,\alpha \right)≜\iint_{x,y}f\left(x,y\right)\exp\left(-2jkx\cos\alpha -2jky\sin\alpha \right)dxdy$$

It can be seen from Equation (4) that F(k,α) is the spectral domain representation of f(x,y) in polar coordinates. Then Equation (3) becomes:

(5) $$S\left(k,\varphi \right)=\int_{-\pi }^{\pi }F\left(k,\alpha \right)\exp\left(2jkR_{0}\cos\left(\varphi -\alpha \right)\right)d\alpha$$

one can note that Equation (5) represents a circular convolution on the variable φ. Consequently, Equation (5) can be written as:

(6) $$S\left(k,\varphi \right)=F\left(k,\varphi \right){*}_{\left(\varphi \right)}\exp\left(2jkR_{0}\cos\varphi \right)$$

where ${*}_{\left(\varphi \right)}$ represents the circular convolution upon variable φ. We know from the properties of discrete Fourier transform (DFT) that the convolution can be actualized by multiplication in the spectral domain. Thus, the following equation can be obtained by doing FFT on both sides of Equation (6):

(7) S(k,ω)=F(k,ω)·H(k,ω)

where $H\left(k,\omega \right)=FFT_{\left(\varphi \right)}\left[\exp\left(2jkR_{0}\cos\varphi \right)\right]$. In Equations (6) and (7), φ,ω are conjugate variables of the Fourier transform.

Following are the steps devised for the proposed imaging according to the derivation:

- **Step one**: Implement FFT to the φ domain of the raw data S(k,φ) to get S(k,ω).
- **Step two**: Generate $\exp\left(2jkR_{0}\cos\varphi \right)$ and implement FFT to its φ dimension to obtain H(k,ω).
- **Step three**: Multiple S(k,ω) by H(k,ω)’s inverse filter $H^{*}\left(k,\omega \right)$ to get F(k,ω).
- **Step four**: Implement IFFT to the ω dimension of F(k,ω) to obtain F(k,α).
- **Step five**: Transform the polar format data F(k,α) into Cartesian coordinates and reconstruct f(x,y) using IFFT.

**Figure 2:**
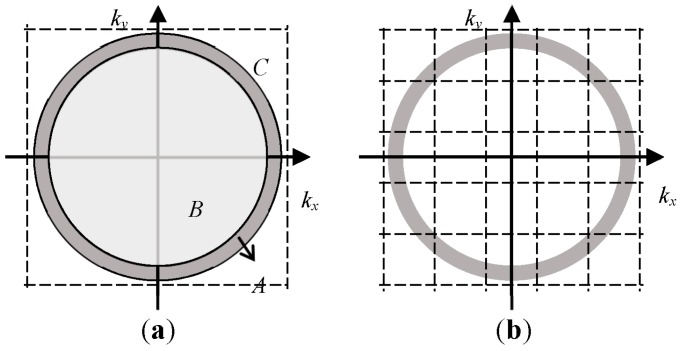
Schematic diagram of the pre-processing procedure for NUFFT (**a**) the spectral before pre-processing; (**b**) the spectral after pre-processing.

When the data volume is large, the interpolation operation in step five will consume a large amount of computing resources. In order to improve the computational efficiency, we adopt the fast Gaussian gridding (FGG)-based NUFFT algorithm to achieve the fast calculation of step five [26]. It should be pointed out that the direct use of NUFFT still faces the problem of a heavy computational load as explained in Figure 2a. According to the properties of Fourier transform, if the ambiguity range of the image is demanded to be L, the sampling interval of the spectral domain Δk should satisfy:

(8) $$\Delta k<\frac{2\pi }{L}$$

The ring region A is the support region in the spectral domain of the echo signal. In the terahertz regime, shape A is an annular region with a large radius but a narrow width. If NUFFT is implemented directly to the polar format data, the region within the dashed box will all be interpolated. However, only the computation within region A which contains the echo signal is meaningful. As region B and C take up the most space within the interpolated area, a great deal of the computing resources are consumed uselessly. Therefore, to improve the NUFFT’s efficiency, the polar format data are pre-processed in the manner shown in Figure 2b. The spectral domain is evenly divided into a number of sub-regions by the dotted line. The interval of the two adjacent dotted lines, K, is determined by the size of the resolution cell Δl in the image domain:

(9) $$K>\frac{2\pi }{\Delta l}$$

Then translate and superpose all the data in each sub-region into one sub-region, and the support region becomes a folded annular shape. This time, as the support region will take up the most space of the interpolated area, the computational efficiency of the FGG manipulation will be significantly improved. 

## 3. Point Targets Simulations

The simulation parameters in this section are listed in Table 1. According to [27] and Table 1, we can conclude that the distance range within which the spherical correction is necessary is 200 m for ideal point targets. This 200 m constraint is also valid for more general targets in the following Experimental Section, although such looser (shorter) constraint exists.

### 3.1. Comparison of Imaging Results before and after Considering Spherical Wave Effect

**Figure 3:**
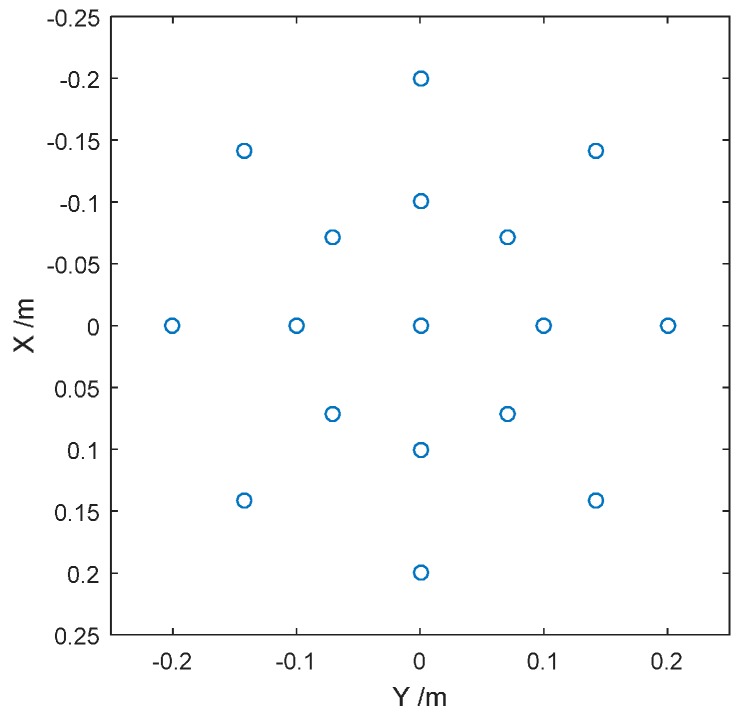
Illustration of the point targets’ location.

In order to explore the influence of the spherical wave on the imaging, we set a number of ideal point targets in different positions in the ROI as shown in Figure 3. 

**Figure 4:**
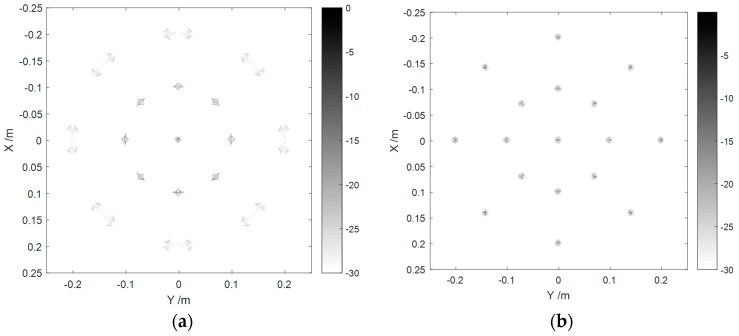
Comparison of imaging results (**a**) before and (**b**) after spherical wave correction.

Figure 4 draws the imaging results before and after the spherical wave correction. As can be seen, under the current parameter configurations, the wavefront curvature dramatically damaged the focusing. It also indicated that the compensation method is correct and effective. We can get the following conclusions from Figure 4: (1) The closer the targets are to the edge of the image, the more obvious are the spherical wave effects that can be observed. The point located at the origin will not be impacted by the wavefront curvature; (2) The defocusing effects are different from point to point, which means the spherical wave effects vary spatially.

### 3.2. Comparison of Imaging Quality

**Figure 5:**
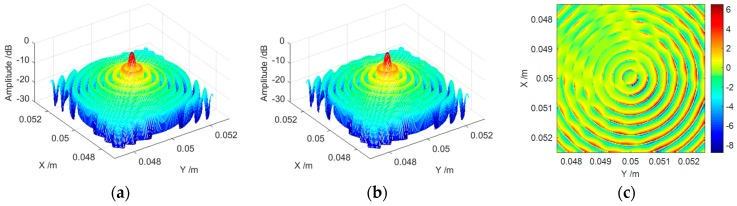
Comparison of PSF obtained by (**a**) CBP; (**b**) our method; and (**c**) the subtraction of (**b**) from (**a**).

In order to validate the accuracy of the spherical wave compensation and the image reconstruction, we take the CBP algorithm as a benchmark standard. This CBP algorithm is of high precision as well as strong flexibility, and the influence of the spherical wavefront can be easily added into the reconstruction procedure. As previously mentioned, the origin point will not be impacted by the wavefront curvature. Thus, we intentionally choose a point target located at (0.05 m, 0.05 m). Figure 5 shows the reconstructed point spread function (PSF) of both methods, and we will see that sub-millimeter spatial resolutions are obtained.

We find it is difficult to distinguish the two PSFs in an eye-inspection manner. In addition, we draw the subtraction of Figure 5b from Figure 5a in Figure 5c. From Figure 5c, we can note that the difference between the two mainlobes and each order’s sidelobes are close to 0 dB which means the reconstruction result of our method is very close to that of the CBP algorithm. Besides, we find that the subtraction between the mainlobe and the first-order sidelobe or between different orders’ sidelobes can reach more than 6 dB. This is caused by the following reasons: firstly, the spatial grids of Figure 5a,b can be slightly different since CBP is a time domain method while our method is a frequency domain method; secondly, the slopes between these lobes are relatively large which means a small horizontal shift can lead to a large difference in the vertical direction. Nevertheless, the relatively large difference of these regions does no damage to the conclusion that Figure 5b is very similar to Figure 5a. 

Further, the peak-sidelobe rate (PSLR) is used to quantitatively analyze the performance of both methods. The result is: the PSLR of Figure 5a is 7.8 dB while that of Figure 5b is 7.9 dB. The reconstruction quality of our method is slightly higher than that of the CBP algorithm, which indicates that the interpolation operation in CBP during reconstruction introduces some errors; on the other hand, it also shows that our method is of high accuracy. 

## 4. Experimental Results

### 4.1. Imaging Results

**Figure 6:**
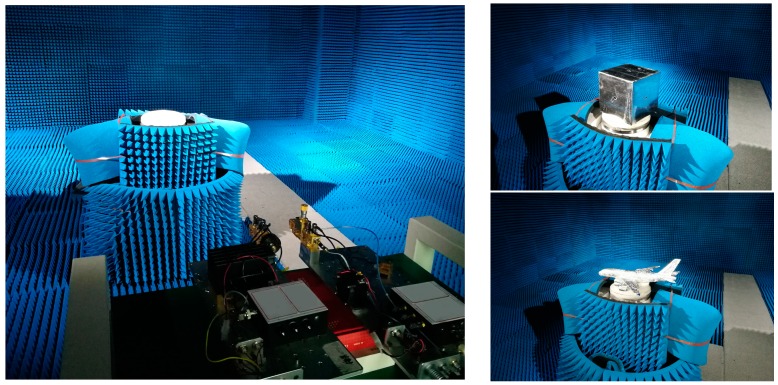
Experimental scenarios.

Figure 6 shows the experimental scenarios. In the experiments, for the sake of obtaining an entire beam illumination of the target as the antenna beam angle is limited, we adjust the $R_{0}$ to 1.5 m. The other parameters are the same as those listed in Table 1.

**Figure 7:**
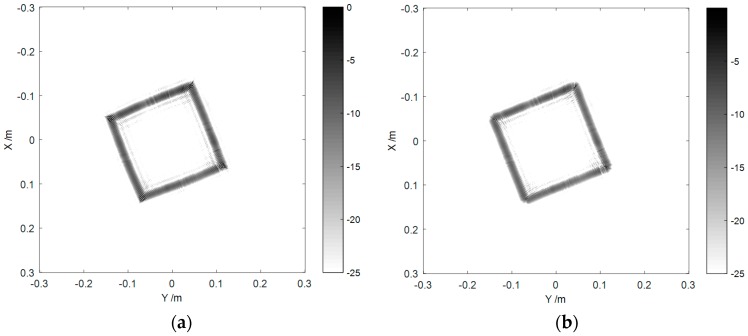
Imaging results of the metal cube (**a**) before and (**b**) after spherical wave compensation.

**Figure 8:**
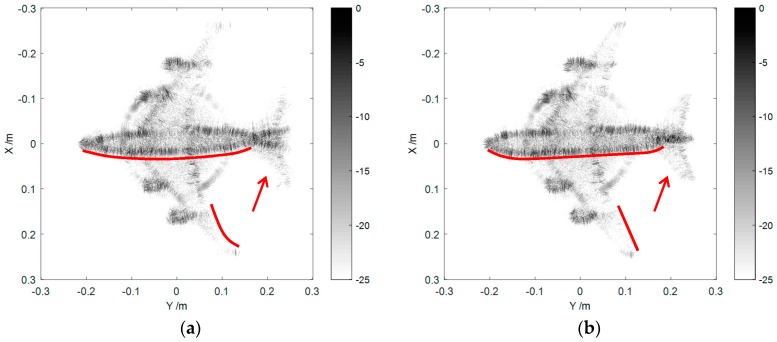
Imaging results of the aircraft model (**a**) before and (**b**) after spherical wave compensation.

As shown in Figure 6, we carried out a near-field measurement on a metal cube and an aircraft model. Their imaging results are drawn in Figure 7 and Figure 8, respectively.

We can see from Figure 7a that due to the influence of the spherical wavefront, the cube edges were bended in the image, which did not match the actual shape. Figure 7b shows the compensated result where the edge bending was calibrated, and the size and shape of the imaging result were highly consistent with the real target. Comparing Figure 4b and Figure 7b, we find that the resolution of Figure 7 is lower than that of Figure 4. This is a natural phenomenon for non-ideal-point targets and can be explained. The cube is a target of highly anisotropy scattering characteristics. Therefore, the radar could only receive the backscattered waves of each edge in few aspects within 360°. This fact confines the 2D resolution of the edges, and it can be deduced that the width of the edges in Figure 7b is determined by the range resolution (or the signal bandwidth).

As can be seen in Figure 8a, obvious distortion and defocusing were observed in the parts close to the edge of the image, such as the ends of the aircraft wing, the tail and the nose. The whole aircraft body was also distorted. In Figure 8b, the imaging result and the actual model were in excellent agreement after the compensation. Besides, comparing Figure 7 and Figure 8, we can find an extra ring-shape target behind the aircraft in Figure 8. This phenomenon was caused by the scattering component from the edge of the circular turntable. As the RCS of the metal cube is much larger than that of the aircraft model, the turntable is suppressed in Figure 7 with the same dB display range as in Figure 8. From the knowledge of targets’ scattering characteristics, we know that the radius of the ring in Figure 8 is about 12 cm and is equal to the practical radius of the plastic disc. 

### 4.2. Computational Complexity Analyses

For a more comprehensive evaluation of our method, the following shows the comparison on the computational complexity to the CBP algorithm. The raw echo data is supposed to be a $N_{f}\times N_{\varphi }$ matrix where $N_{f},N_{\varphi }$ represents the number of samplings of the frequency and the azimuth dimension, and $N^{2}$ denotes the number of pixels in the image. The main steps and computational complexity of our method and the CBP are shown in Table 2 and Table 3, respectively.

**Figure 9:**
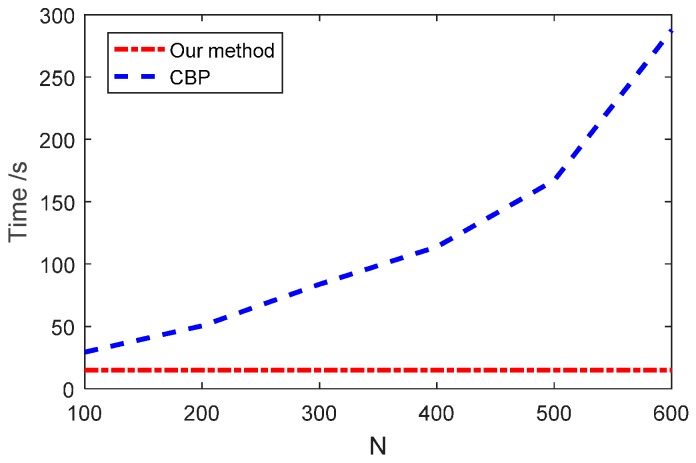
Time costs comparison.

Generally speaking, the larger the $N_{f}\times N_{\varphi }$ is, the larger the $N^{2}$ is. Therefore, we can suppose that $O\left(N_{f}N_{\varphi }\right)$ is in the same order as $O\left(N^{2}\right)$. Accordingly, we can simply do a rough comparison and find that the computational complexities of the two methods are $O\left(N^{2}\log N\right)$ and $O\left(N^{3}\right)$, respectively. In order to exhibit the advantages of our method on computational efficiency more directly, the time costs coping with the same experimental data are recorded against N of both methods. The results are plotted in Figure 9. The data are obtained on a HP work station Z820 with Intel Xeon CPU E5-2670 using Matlab codes (Hewlett Packard Company, Palo Alto, CA, USA).

As shown in Figure 9, the time cost of our method hardly increased with the increase of N and it held the line at about 15 s, while that of CBP increased dramatically. It indicated that the main computational time of our method was from the FGG procedure. We think that Matlab has paralleled the IFFT operation. Therefore, the time cost of IFFT which should have increased with N can be neglected compared to the time cost of the FGG operation.

## 5. Conclusions

We studied the terahertz wide-angle fast imaging method at a relatively short distance where spherical effects cannot be ignored. The spherical wavefront correction was done by a circular convolution of the rotation angle φ which can be accelerated by the FFT technique. Meanwhile, to improve the efficiency of the image reconstruction, we introduced the FGG-based NUFFT technique to wide-angle focusing. According to the simulation results of the point targets, we can conclude that: the closer the targets are to the edge of the image, the more obvious the spherical wave effects that can be observed; and the spherical wave effects are spatially variable. By comparing the imaging results of the experimental data to that of the CBP algorithm, the effect of the spherical compensation and the efficient of the reconstruction can be verified. The experimental results also show that the focused images are of high spatial resolution under circular measurement conditions. Our method can be directly used for nondestructive detection or safety inspections. It also provides a feasible way to calculate the far-field RCSs using near-field data in the terahertz regime.

## Figures and Tables

**Table 1:** Parameters configuration.

$R_{0}$	$\varphi_{\max}$	Δφ	Center Frequency	Bandwidth	Region of Imaging (ROI)
1 m	360°	0.0225°	220 GHz	12.8 GHz	0.5 × 0.5 m^2^

**Table 2:** Computational complexity of our NUFFT-based spherical compensation and reconstruction method.

Operations	Computational Complexity
Spherical wavefront compensation	$O\left(N_{f}N_{\varphi }\log N_{\varphi }\right)$
FGG [26]	$O\left(N_{f}N_{\varphi }\right)$
2D-IFFT	$O\left(N^{2}\log N\right)$

**Table 3:** Computational complexity of the CBP algorithm.

Operations	Computational Complexity
FFT in range direction	$O\left(N_{\varphi }N_{f}\log N_{f}\right)$
Spherical interpolation and coherent summation along azimuth angle	$O\left(N_{\varphi }N^{2}\right)$
